# Functional outcome prediction of ischemic stroke patients with atrial fibrillation accepting post-acute care training

**DOI:** 10.3389/fneur.2022.954212

**Published:** 2022-09-23

**Authors:** Sen-Yung Liu, Ying-Lin Hsu, Yi-Chun Tu, Ching-Hsiung Lin, Shih-Chun Wang, Ya-Wen Lee, Yin-Tzer Shih, Ming-Chih Chou, Chih-Ming Lin

**Affiliations:** ^1^Institute of Medicine, Chung Shan Medical University, Taichung, Taiwan; ^2^Department of Rehabilitation Medicine, Changhua Christian Hospital, Changhua, Taiwan; ^3^Department of Applied Mathematics and Institute of Statistics, National Chung Hsing University, Taichung, Taiwan; ^4^Institute of Data Science and Information Computing, National Chung Hsing University, Taichung, Taiwan; ^5^Division of Chest Medicine, Department of Internal Medicine, Changhua Christian Hospital, Changhua, Taiwan; ^6^Institute of Genomics and Bioinformatics and Ph.D. Program in Translational Medicine, National Chung Hsing University, Taichung, Taiwan; ^7^Department of Recreation and Holistic Wellness, MingDao University, Changhua, Taiwan; ^8^Department of Medicine Imaging, Changhua Christian Hospital, Changhua, Taiwan; ^9^Department of Nursing, Hanming Christian Hospital, Changhua, Taiwan; ^10^Division of Thoracic Surgery, Department of Surgery, Chung Shan Medical University Hospital, Taichung, Taiwan; ^11^School of Medicine, Chung Shan Medical University, Taichung, Taiwan; ^12^Department of Family and Community Medicine, Chung Shan Medical University Hospital, Taichung, Taiwan; ^13^Graduate Institute of Statistics and Information Science, National Changhua University of Education, Changhua, Taiwan; ^14^Department of Neurology, Changhua Christian Hospital, Changhua, Taiwan; ^15^Department of Post-baccalaureate Medicine, College of Medicine, National Chung Hsing University, Taichung, Taiwan

**Keywords:** ischemic stroke, atrial fibrillation, Barthel Index, NIHSS, mRS, post-acute care, plaque index, EQ-5D

## Abstract

**Background:**

Ischemic stroke poses a major threat to human health and represents the third leading cause of death worldwide and in Taiwan. Post-acute care (PAC) training has been reported to be beneficial for post-index stroke events. However, knowledge is still lacking on the outcome of stroke events with cardiac origin. The focus of the current study is to investigate the effectiveness of PAC in this subgroup of patients as well as identify key baseline pointers that are capable of early prediction of patients' physical recovery. In addition, the authors hypothesize that the routinely arranged non-invasive carotid duplex that evaluates the characteristics of the carotid lumen could play a significant role in providing an early outcome prediction.

**Methods:**

For the current research, 142 ischemic stroke patients with underlying cardiac arrhythmia (atrial fibrillation) were retrospectively recruited. The patients' basic demographics, neuroimaging, carotid duplex, and basic biochemistry datasets were accurately documented. The pre and post-admission National Institutes of Health Stroke Scale (NIHSS) (6-month follow-ups), Barthel Index, and mRS score (12-month follow-ups) were also recorded. All statistical analyses were performed using R for Windows (version 3.6.3). Barthel Index, NIHSS, and mRS scores obtained before and after hospitalization were compared to determine the patients' outcomes and were classified as improved or unimproved. A multivariate logistic analysis was designed and applied to assess the significance of risk factors and to obtain the odds ratios (ORs). The receiver operating characteristic (ROC) curve and the Youden Index was used to find the important cut-off point information, and the area under the curve (AUC) was calculated to provide accuracy.

**Results:**

The average age of the 142 ischemic stroke patients enrolled in the current study was about 66 years, of which 88 patients were male and 54, female. Many of them had other comorbidities: 86 patients had mixed hyperlipidemia (60.56%), 115 had hypertension (80.99%), and 49 suffered from diabetes mellitus (34.51%). The mRS showed an improvement in the condition of only 40 patients (28.175%), whereas the Barthel Index showed improvement in 71 patients (50%), and 68 patients (47.89%) showed recovery on the NIHSS. The Barthel Index and NIHSS were selected because they already had an almost equal number of samples among the improved and unimproved groups (50%), rather than mRS, which had a lower number (28.17%) of improved cases. While conducting the EuroQol-5 Dimension (EQ-5D) assessment, anxiety/depression stood out as the most prominent issue, affecting 44 patients (30.99%). Self-care was another factor that was involved in the ongoing improvement of 36 patients (25.35%). Multivariate logistic analysis of both NIHSS and Barthel Index showed improvement with a contralateral plaque index statistical significance (P<0.05), whereas NIHSS showed a relevant significance in anxiety/depression and Barthel Index registered usual activity in the data analysis (P<0.05). ROC curve and Youden index analysis showed similar results in both NIHSS and Barthel Index of contralateral plaque index of 4.5, this being the cutoff point value for this group of patients.

**Conclusion:**

In the current study, nearly half of the enrolled patients showed favorable functional recovery. The outcome assessments seem to correlate well with NIHSS and Barthel Index scores, rather than mRS. The anxiety/depression and usual activities domains of the EQ-5D results are associated with and have a great impact after the patients undertake the PAC rehabilitative strategy. Moreover, the variables obtained through carotid duplex and plaque index might also play a significant role in determining the patient's functional outcome.

## Highlights

- Post-acute care strategy for ischemic stroke patients with underlying cardiac arrhythmia is important and its effectiveness can be seen in NIHSS and Barthel Index assessment tools.
- The program presented in our hospital and affiliated branch facility is capable of ensuring (and increasing the willingness) of ischemic stroke patients' rehabilitative participation leading to safe and satisfactory long-term neurological sequelae recovery.
- The mood swing, particularly post-stroke anxiety/depression, as well as daily life usual activities improvement monitored through EQ-5D testing, have a significant impact in terms of intermediate and long-term stroke functionality recovery.
- The non-invasive and routinely obtained extra-cranial carotid ultrasound for ischemic stroke patients seems to play a crucial role in the differentiation of patients' outcome.
- The characteristics of the extra-cranial carotid systems assessed by the carotid ultrasound and plaque index can provide first-line clinicians with easy and non-complex clinical information for decision-making and early stroke outcome prediction.

## Introduction

Ischemic stroke is the third leading cause of mortality and morbidity worldwide and in Taiwan (1). Several factors lead to the occurrence of a stroke and this study focuses on cardiogenic causes. A patient can suffer from cardiogenic arrhythmia and atrial fibrillation (2), a type of comorbidity that occurs often among elderly people, but frequently ignored. The initial manifestation of this type of stroke is not necessarily obvious and without carrying out meticulous analysis of both the cerebral and cardiogenic areas using cerebral magnetic resonance imaging and 24-h Holter monitoring, it can often be misdiagnosed as a common cold or stress. Most of the published literature focuses on the acute/super acute treatment of ischemic stroke through intravenous thrombolytic and/or intra-arterial thrombectomy (IA) (1–3) bridging therapy as well as the satisfactory results following post-acute care (PAC) (4–6). However, there is a clinical gap in information on the outcome of ischemic stroke patients with atrial fibrillation after PAC training. Cardiogenic induced strokes have a greater tendency to reoccur within 3 months of the post-index event. Therefore, special rehabilitative training is required to avoid recurrent strokes. This subgroup of patients accounts for 20–25% of the ischemic stroke patient population (6), and if left unintended, it could lead to severe permanent neurological sequelae and create more economic pressure for both the family and society in the long run. In Taiwan, post-acute care (PAC) programs have been endorsed by the Taiwanese government mainly to divert the heavy load of patients from overcrowding tertiary medical facilities (medical centers) for mild diseases. When the patient's condition becomes less severe but long-term intense rehabilitative personnel and facilities are still required, this initiative encourages the patient to get more involved in the training program to speed up the recovery process (7). According to the government policy, survivors of stroke, traumatic brain injury and burns, geriatric and multiple systemic failure patients, and persons with bone fractures can be admitted to this kind of program. Among these, ischemic stroke patients constitute the largest number, because their rehabilitation often needs more intense and long-term training with both physical and psychological follow-ups. In the current study, we intended to understand if ischemic stroke patients with underlying atrial fibrillation receiving the intense rehabilitative program showed favorable long-term outcomes as well as to define various outcome assessment projections. In addition, we aimed to identify potential baseline variables that could be linked to the patients' discharged condition and early prediction of patients' outcomes.

Secondly, during the intense rehabilitative program at PAC hospital, enrolled patients were given various questionnaires. Among them, the EQ-5D (8) was the most popular and was recognized as the most effective way to detect if patients were experiencing any sort of emotional discomfort or internal pain. Given this, this study aimed to see if the items of EQ-5D could be integrated into outcome assessment tools such as the mRS, Barthel Index, and NIHSS scores. Often, pain and depression are neglected in the clinical setting as most front-line neurologists pay more attention to the onset of new neurological symptoms, particularly signs of a possible new onset of stroke or deterioration. The carotid duplex is a non-invasive, inexpensive, and mobile monitoring clinical apparatus that enables the doctor to monitor the patient's cerebral blood flow in real time. This exam is routinely arranged for stroke patients and is carried out during the first few days of the acute neurological ward admission. Given the convenience of this monitoring tool, this study aimed to define potential carotid duplex-derived parameters to predict the patient's outcome early and associate it with outcome assessments. We also sought to identify useful cut-off points and clinically important information to support the clinicians' decision-making processes. For instance, we hypothesize the characteristics of the carotid lumen condition (plaque formation or number of plaques in the carotid systems) might play a significant role in this regard. This study advocates the importance of the PAC for stroke patients and provides evidence-based analysis for the use of carotid duplex in clinical practice.

## Materials and methods

### Study protocol and patient selection

In this investigation, we retrospectively studied the data of 200 ischemic stroke patients with underlying cardiogenic atrial fibrillation. This was made possible by the collaboration between the Department of Neurology at Changhua Christian Hospital (CCH) and the Department of Internal Medicine at Hanming Christian Hospital (HCH), through the collection of patients' datasets and medical records between January 2018 and February 2020. The initial stroke patients' information was collected at CCH and the patients were transferred to HCH for continued intense rehabilitative training. Continued datasets were also documented at HCH and integrated with the total of the patients' basic demographics, biochemistry, outcome assessments, and neuroimaging studies. Carotid duplex datasets were manually collected and fully recorded in Excel files. The complete datasets were stored in the study room of basement 1 at the Department of Nursing and one case manager was responsible for keeping the datasets in a secure place. The initial 200 patients were screened, and their number was later reduced to a final number of 142 participants, according to predefined inclusion and exclusion criteria. All the patients were enrolled through the triage in the Emergency Department and evaluated by the stroke neurologist to not be considered thrombolytic and/or intra-arterial thrombectomy treatment candidates. As a general practice, the cardiac arrhythmia of atrial fibrillation /flutter would be confirmed either by the ECG and/or previous medical history and a cardiologist would be consulted for final validation. The participants were subsequently admitted to the neurological ward for advanced treatment for the acute phase of the stroke situation. After the acute phase was stabilized, the post-acute care (PAC) program (9, 10) was explained by the stroke case manager at CCH and the patients then agreed to be transferred to HCH for a continuous rehabilitative program.

The stroke patients stayed at HCH for training before being discharged from the ward care and then would return to outpatient clinics for follow-ups of up to 12 months (1 year or above). The clinical information collected was cross-checked to ensure consistency between the original (paper) medical records and the electronic information of the Changhua Christian Hospital 2000 computer-based medical record systems network, both at CCH and HCH, to ensure the accuracy and consistency of the datasets.

The study protocol was approved by the Research Ethics Committee at Changhua Christian Hospital (Refer to IRB, certificate number: 210713). Informed consent was waived as personal information of the patients was removed from the raw datasets and no potential breach was made beforehand.

All patients included in the study met the following pre-defined inclusion and exclusion criteria: (1) were aged between 20 and 90 years, with evidence of ischemic stroke episode at the index event and evaluated by the cerebral computed tomography (CT), and/or angiography (CTA), and CT perfusion (P), and cerebral magnetic resonance imaging not suitable for intravenous thrombolytic therapy and/or intra-arterial thrombectomy treatment; (2) the cardiogenic arrhythmia atrial fibrillation/flutter was confirmed by an ECG and/or 24-h cardiologic Holter monitoring, and/or previous past medical history and medication documentation; (3) no observation of recurrent cerebral or other vascular events during the study period; (4) the etiology of the current index stroke episode was evaluated and validated to be attributed to the cause of cardiogenic origin, excluding other contributing sources, patients with no severe carotid stenosis/vertebral stenosis having ever received carotid endarterectomy or carotid stenting and intracranial basal vasculature severe stenosis was confirmed by the magnetic resonance imaging studies. Other subtypes/etiologies of stroke mechanism were excluded, for example, internal medicine-induced or medication-contributed; (5) completion of at least 12 months of follow-up after treatment. The exclusion criteria were: (1) NIHSS score <4 or >25 points at the initial evaluation, and/or recurrent stroke of the index stroke episode; (2) patients with cerebral bleeding based on the cerebral tomography scan; (3) patients with cerebral arteriovenous malformations or aneurysms; (4) recurrent stroke during the study period; (5) patients demonstrating systemic vascular events or recurrent cerebral vascular episodes.

Baseline biochemistry and neuro-radiological exams were carried out in the emergency room and neurological ward. The comorbidities, neurological and physical exams, and relevant previous drugs and personal history were carefully recorded during the hospitalization period. The carotid duplex test was conducted within 3 days of initial admission to the hospital. All the parameters of the carotid ultrasound were documented.

The patient's stroke severity was recorded using the NIHSS score (11), Barthel Index (12), and mRS (13) and documented both upon admission to the ward and outpatient clinic follow-ups. NIHSS was repeated for 6 months after neurological ward discharge while Barthel Index and mRS were repeated until 12 months. The mRS was evaluated upon admission to the Department for Emergency at CCH and reevaluated after the patients had been discharged from the HCH and continued during the 3, 6, and 12-month follow-ups at the outpatient clinics. Barthel Index was initially checked during admission to the CCH neurological ward and was followed up again after the patients left HCH and again at the outpatient clinics up to 12 months later. NIHSS score was documented upon admission to the Department of Emergency of CCH and followed up once more after the patients had been discharged and at outpatient clinics for up to 6 months. Based on these predefined categories, we subjectively defined the outcome assessment tools; NIHSS for intermediate follow-up, Barthel Index, and mRS for long-term functional assessment tools.

The modified Rankin Scale (mRS) is a commonly used scale for measuring the degree of disability or dependence in the daily activities of people who have suffered neurological disability from a stroke or other causes. The scale runs from 0 (perfect health without symptoms) to 6 (death). The NIHSS score specifically measures the patients' neurological function and runs from 0 to 42 points, where the higher the points, the more unfavorable/worse the neurological deficits can be. The Barthel index is a clinical assessment tool designed for gauging the capacity of daily life activity ranging from 0 to 100 points, with the highest points representing the best daily independence a patient can reach. The information collected may improve the ability to prognosticate the outcome. Each assessment was conducted by two physicians and any discrepancies were resolved in consultation with a third physician/ case manager for final validation.

The post-acute care (PAC) program is a special program designed for those patients who need an intensive or continuous/extended training rehabilitative program. Under current Taiwanese government regulations, only those afflicted by stroke, burns, bone fractures, traumatic brain injury, or geriatric patients with multiple underlying diseases, fit the governmental reimbursement plan and can be supported by the Bureau of Health Ministry (14, 15). As stroke patients often require long-term or specific training programs, most of them, after being admitted at CCH, would then be transferred to HCH for their therapies. As previously stated, CHH and HCH are connected, therefore the doctors from CCH can also visit and have regular clinics at HCH. PAC runs from Monday to Friday. Every day there are two sessions of 3-h training duration each depending on the specific therapeutic needs of each patient, be it speech training, upper or lower extremities training, or fine motor skills recovery. Patients are to be treated at HCH for six weeks. During this training period, adhering to the program, we also applied the functional oral intake scale (FOIS) (16) to evaluate the gravity of a potential swallowing problem (dysphagia) that could afflict post-stroke patients; carried out EQ-5D (8) questionnaires to measure the post-stroke health-related quality of life; performed mini nutritional assessment (MNA) (17), and tested the capability of carrying instrumental activities of daily living (IADL) (18, 19). The above-mentioned testing batteries and evaluations have been thoroughly documented and placed as the baseline variables to correlate the outcome assessment tools (NIHSS, Barthel Index, and mRS), to see if the short-term training and evaluations could have projected the long-term and well correlation (refer to Appendix Tables A1–A5 in Supplementary material). FOIS is a clinical evaluating tool that is to test oral intake ability, with level 7 indicating the full swallowing ability and level 1 the poorest ability and the need for additional assistance for food intake. This part of the examination is often conducted by a speech therapist at HCH, and the values are documented daily to see the improvement during hospitalization. EQ-5D is a standardized measure of health-related quality of life developed by the EuroQol Group (8) to provide a simple, generic questionnaire to use in clinical and economic appraisal and population health surveys. The questionnaire is carried out by healthcare professionals to assess the physical and emotional components of a patient's condition. There are categories of mobility, self-care, usual activity, pain/discomfort, and anxiety/depression, where the highest points represent the poorest functionality. On the other hand, MNA is a simple test that could easily evaluate patients' nutritional state during hospitalization, and it is usually assessed by clinical dieticians. Its scoring can be divided into: <17, indicating a poor nutritional status and malnutrition; 17–23.5, an acceptable condition but still mild malnutrition, 24–30, suggesting a good and normal nutritional status. IADL is a clinical evaluating tool for the independent functionality required to carry out more complex daily chores. There are eight aspects to be evaluated, such as shopping for groceries, self-medication, the use of mobile phone devices, doing laundry, traveling/commuting, dealing with family affairs, financing, and food preparation. The higher the points, the more normal the patient can function.

### Cervical carotid duplex exam

All procedures were conducted by a stroke neurologist team in a specialized angiography clinic at Changhua Christian Hospital. Cervical carotid artery (CCA) ultrasound examinations (Philips iE33 7-Mhz linear transducer) were performed on patients after arriving at the emergency room. Cross-sectional B-mode scanning was performed to detect intraluminal plaque material and the longitudinal screening method was adopted to confirm the presence of plaque. Two physicians assessed and classified plaques into subtypes 1, 2, 3, or 4, according to the International Classification System (20). Whenever there was a disagreement between the physicians, a third physician would make the final assessment. The intima-media thickness of the mid-portion of the CCA was measured on the ipsilateral side of the index stroke event. The parameters of peak systolic velocity (PSV), end-diastolic velocity (EDV), resistance index (RI) [calculated as (PSV-EDV)/PSV], and pulsatility index (PI) [calculated as (PSV-EDV)/mean of the velocity] of the common carotid artery (CCA), internal carotid artery (ICA), external carotid artery (ECA), vertebral artery (VA), and ophthalmic artery (OA) were measured bilaterally. The reversal of blood flow in the OA was also measured. The forward flow was defined as blood flow detected out of the stenotic ipsilateral carotid artery, whereas reverse flow was defined as blood flow into the carotid artery (21, 22). The machine Philips iE33 7 automatically calculated the plaque index (23) on both the ipsilateral and contralateral sides of the cerebral lesion.

### Cerebral computed tomography/angiography and/CT perfusion

**Figure 1:**
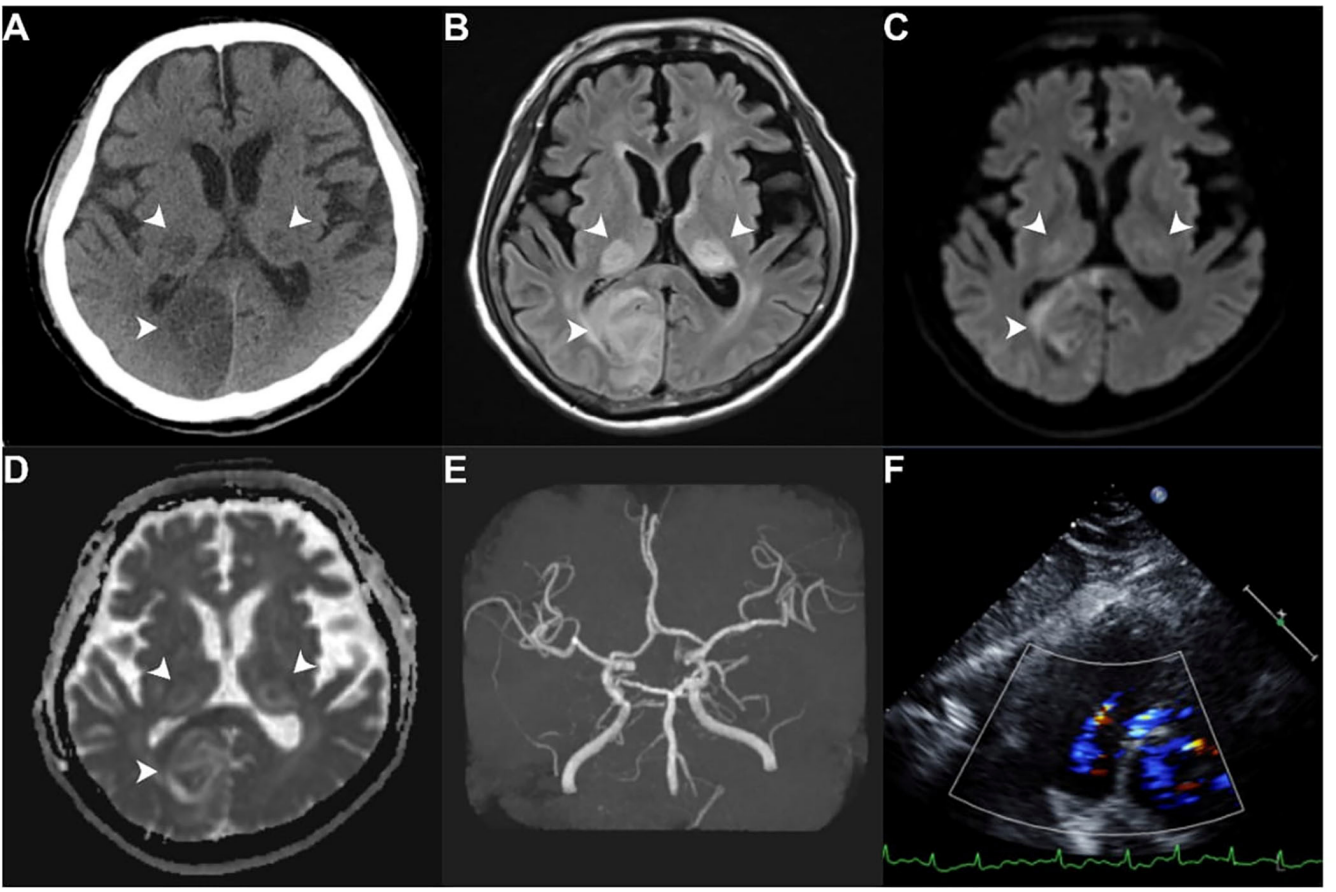
A stroke patient with atrial fibrillation. A 71-year-old male with a history of atrial fibrillation presented with altered mental status. He was admitted to the neurological ward due to a suspected ischemic stroke event. Head computed tomography without contrast revealed hypodense areas in bilateral thalami and occipital lobes [white arrowhead **(A)**]. Head magnetic resonance imaging confirmed acute cerebral infarction [white arrowhead **(B–D)**]. Magnetic resonance angiography reported a generalized atherosclerotic change of the bilateral posterior cerebral artery and severe narrowing of the right proximal carotid siphon **(E)**. Transthoracic echocardiography showed a normal left ventricular ejection fraction of 57% **(F)**. **(A)** Head computed tomography without contrast; **(B)** T2FLAIR magnetic resonance imaging; **(C)** Diffusion-weighted magnetic resonance imaging; **(D)** Apparent diffusion coefficient magnetic resonance imaging; **(E)** Magnetic resonance angiography; **(F)** Four chamber echocardiographic views of echocardiography.

Multiple sequential axial images were obtained from the skull base up through the vertex without intravenous contrast material. The technical parameters were thickness, 5 mm; length, 200 mm; increment, 10 mm; kV, 120; mA, 550. CTA examinations were performed using a second-generation dual-source CT scanner (SOMATOM Definition Flash, Siemens Healthcare, Forchheim, Germany). After placing an 18-gauge intravenous catheter in the antecubital vein, 100 ml of Iodixanol (Visipaque 320, GE Healthcare, Carrigtwohill, Ireland) or Iohexol (Omnipaque 350, GE Healthcare, Ireland) was infused as the contrast medium at a rate of 5 ml/s. The initial injection delay was estimated using the bolus-tracking technique, where the threshold was 100 Hounsfield units. Scanning was performed using a dual-energy mode with pitch, rotation time, and collimation of 0.9, 0.28 s, and 2 mm × 32 mm × 0.6 mm, respectively, at 100 kV/150 ref mAs (Tube A) and Sn140 kV/178 ref mAs (Tube B). The area from the aortic arch to the top of the neuro-cranium was scanned. CT images were reconstructed at a slice thickness of 0.6 mm and increment of 0.3 mm with a medium–smooth kernel. CTP scans were subsequently performed with a contrast bolus of 50 ml of Omnipaque 350 (GE Healthcare, Milwaukee, WI, USA). Perfusion data sets were post-processed using a Siemens Multimodality Workplace Workstation (Siemens Medical, Germany), which calculated the mean transit time (MTT), the cerebral blood volume (CBV), the cerebral blood flow (CBF), and the time to peak (TTP). The arterial input and venous outflow curves were analyzed to ensure data set completeness. A clinical case is depicted in Figure 1.

### Magnetic resonance imaging and angiography

Structural and functional MR imaging and angiographic examinations were performed using a 3-T (Magnetom Verio, Siemens Healthcare, USA) or a 1.5-T imager (Magnetom Aera, Siemens Healthcare) with a cervical coil. The standard protocol for evaluating a stroke including axial diffusion-weighted imaging (DWI), apparent diffusion coefficient, and fluid-attenuated inversion-recovery sequences were followed. Three-dimensional TOF MR angiography without contrast enhancement was performed in the transverse plane using a sliding interleaved kY acquisition sequence comprising 6 overlapping slabs of 11 sections, with the following parameters: section thickness, 1.2 mm; repetition time (milliseconds)/echo time (milliseconds), 242/7; flip angle, 20°; field of view, 200 × 200 mm; matrix, 205 × 320. The final pixel size was 0. 975 mm × 0.625 mm. The entire imaging time was ~7 mins. A contrast-enhanced MR angiography was not routinely performed (Figure 1).

## Statistical analysis

The research is based on the data from Changhua Christian Hospital and Hanming Christian Hospital regarding 142 acute stroke patients with atrial fibrillation who received high-intensity rehabilitation. Numerous variables were considered such as individual outcome assessments, personal information, previous disease, biochemistry, carotid duplex sonography, and neuroimaging.

All statistical analyses were performed using R for Windows (version 3.6.3). The significance level was set at *P* < 0.05 for all analyses. Before and after hospitalization, mRS, NIHSS, and Barthel Index scores were compared to determine patient outcomes and classified as improved vs. unimproved groups.

To test the association between the degree of disability and variables before hospitalization, with the degree of disability after PAC, the definitions of the outcome assessment were pre-set as: ΔmRS = Discharge mRS – Admission mRS, <0 defined as improved, ≧0 defined as unimproved; ΔBarthel Index = Discharge Barthel Index – Admission Barthel Index, >0 defined as improved and ≦0 defined as unimproved; ΔNIHSS = Discharge NIHSS – Admission NIHSS, <0 defined as improved; ≧0 defined as unimproved. The paired samples *t*-test was used to compare the means of two measurements taken from the same patient before and after hospitalization. The independent *t*-test was used to compare the mean of the improved and the unimproved groups to determine whether the means were significantly different. Categorical variables were compared using the chi-square test of independence to explore the relationship between outcome assessments (Barthel Index and NIHSS) and baseline variables. Yule's Q (24) was used to calculate the correlation coefficient and reflected the degree of association. Univariate and stepwise multivariate logistic regressions were modeled and used to assess the significance of risk factors and obtain the odds ratios (ORs).

The receiver operating characteristic (ROC) curve was determined to find the cut-off point, and the area under the curve (AUC) indicated the overall accuracy of the degree of disability and the variables. Further, Youden's index (25) was chosen to determine the cut-off point.

## Results

Based on the basic information obtained from the 142 patients, the authors analyzed the datasets into several parts.

Table 1A shows the patients' baseline personal information and routine biochemistry workups. As previously stated, the average age of the patients (88 males and 54 females) was 66 years and among the underlying diseases the most common were mixed type hyperlipidemia (60.56%), essential hypertension (80.99%), and diabetes mellitus type 2 (34.51%). As for personal habits, 38 patients were smokers (26.76%). The patients were treated in Changhua Christian Hospital for 17 days and successively in Hanming Christian Hospital thereafter for 41 days on average.

**Table 1A:** Baseline patients' personal demographic and biochemistry.

**Variables**	***n*** **(%) or median (range)**
**Personal information**	
Age (year)	66 (34–98)
Gender (male)	80 (56.34%)
Height (cm)	160.00 (138.40–183.00)
Weight (kg)	64.30 (34.80–124.00)
BMI	24.72 (15.47–42.39)
SBP (mmHg)	156 (90–240)
DBP (mmHg)	87 (51–140)
Hospital stay in CCH	17 (4–38)
Hospital stay in HCH	41 (1–84)
**Previous disease**	
DM type 2	49 (34.51%)
HTN	115 (80.99%)
Gout	12 (8.45%)
CKD	2 (1.41%)
COPD	2 (1.41%)
CAD	17 (11.97%)
Mixed type hyperlipidemia	86 (60.56%)
Previous stroke	33 (23.24%)
Smoking	38 (26.76%)
Drinking	16 (11.27%)
Betel nut	3 (2.11%)
**Biochemistry**	
HbA1c	6.00 (4.20–15.20)
Ac sugar (before meals)	100.50 (66.00–212.00)
Cholesterol	161.00 (83.00–290.00)
HDL	41.00 (21.00–121.00)
LDL	107.00 (5.80–222.00)
Triglyceride	105.00 (44.00–404.00)
Uric acid	5.20 (2.00–10.40)
Na^+^	138.00 (125.00–152.00)
K^+^	3.80 (2.70–9.70)
GOT	24.00 (13.00–148.00)
GPT	19.00 (6.00–98.00)
Creatinine	0.76 (0.41–11.45)
GFR	87.23 (4.62–192.05)
APTT	30.45 (11.90–53.80)
INR	1.00 (0.18–3.30)

For continuous variables, it shows their median and range (minimum–maximum). For discrete variables, it shows their number and proportion.

CCH, Changhua Christian Hospital; HCH, Hanming Christian Hospital; SBP, systolic blood pressure; DBP, diastolic blood pressure; DM, diabetes mellitus; HTN, Hypertension; CKD, chronic kidney disease; COPD, chronic obstructive pulmonary disease; CAD, coronary artery disease; HbA1c, Glycated Hemoglobin; Ac Sugar, Glucose Ante Cibum (before meals); HDL, high density lipoprotein; LDL, low density lipoprotein; GOT, glutamic oxaloacetic transaminase; GPT, glutamic pyruvic transaminase; GFR, glomerular filtration rate; APTT, activated partial thromboplastin time; INR, international normalized ratio.

Table 1B shows the routine baseline carotid duplex dataset results. For RI (resistance index), it was about 0.75 in every carotid. For PI (pulsatility index), it was about 1.6 in every carotid. For IMT (intima-media thickness), it was about 0.8 on both ipsilateral and contralateral sides.

**Table 1B:** Baseline patients' carotid duplex datasets.

**Variables**	***n*** **(%) or median (range)**
**Carotid duplex sonography and neuroimaging**	
Ipsi CCA RI	0.78 (0.59–1.00)
Contra CCA RI	0.76 (0.57–0.91)
Ipsi ICA RI	0.70 (0.52–1.00)
Contra ICA RI	0.73 (0.44–1.00)
Ipsi ECA RI	0.89 (0.54–1.85)
Contra ECA RI	0.89 (0.65–1.76)
Ipsi VA RI	0.77 (0.55–1.78)
Contra VA RI	0.77 (0.49–1.69)
Ipsi IMT	0.80 (0.40–1.90)
Contra IMT	0.83 (0.42–2.50)
Ipsi Plaque Index	2.00 (0.00–11.00)
Contra Plaque Index	2.50 (0.00–11.00)
Ipsi ICA duplex diameter	4.60 (3.00–7.00)
Contra ICA duplex diameter	4.87 (3.00–7.00)
Ipsi ICA volume	231.00 (5.98–525.00)
Contra ICA volume	64.50 (1.00–130.00)
Ipsi CCA PI	1.69 (0.98–3.73)
Contra CCA PI	1.60 (0.50–3.73)
Ipsi ICA PI	1.25 (0.76–6.14)
Contra ICA PI	1.30 (0.61–3.73)
Ipsi ECA PI	2.25 (0.78–5.98)
Contra ECA PI	2.47 (0.78–5.55)
Ipsi VA PI	1.62 (0.78–5.97)
Contra VA PI	1.65 (0.77–7.03)
MRI Ipsi MCA stenosis	76 (53.52%)
MRI contra MCA stenosis	47 (33.10%)

For continuous variables, it shows their median and range (minimum - maximum). For discrete variables, it shows their number and proportion.

Ipsi, ipsilateral; Contra, contralateral; CCA, common carotid artery; ECA, external carotid artery; ICA, internal carotid artery; VA, vertebral artery; RI, resistance index; IMT, intima-media thickness; PI, pulsatility index; MRI, magnetic resonance imaging; MCA, middle cerebral artery.

Table 1C represents the short, intermediate, and long-term outcome assessments and the EQ-5D results. It shows the three outcome assessments compared in this study. The improvement of just 40 patients (28.175) was observable in the mRS, whereas the Barthel Index showed that 71 patients (50%) had improved, and the conditions of 68 patients (47.89%) showed improvement in the NIHSS. We selected the Barthel Index and NIHSS results which registered a similar number of samples of improved and unimproved groups (50%). In the EQ-5D assessment undertaken during the Hanming Christin Hospital treatment, “anxiety/depression” stood out as the most prominent factor in 44 patients (30.99%), followed by “self-care,” which showed improvement in 36 patients (25.35%) (refer to Appendix Tables A1–A5 in Supplementary material).

**Table 1C:** Outcome assessments and EQ-5D results.

**Variables**	***n*** **(%) or median (range)**
**Outcome assessments**	
ΔmRS	0 (−4 to 3)
mRS improved	40 (28.17%)
ΔBarthel Index	5 (−65 to 100)
Barthel Index improved	71 (50.00%)
ΔNIHSS	−1.00 (−30.00 to 12.00)
NIHSS improved	68 (47.89%)
**EQ-5D**	
Mobility improved	26 (18.30%)
Self-care improved	36 (25.35%)
Usual activities improved	21 (14.79%)
Pain/discomfort improved	29 (20.42%)
Anxiety/depression improved	44 (30.99%)

ΔmRS, discharged mRS – Admission mRS; <0 defined as improved and marked as 1; ≧0 defined as unimproved and marked as 0; ΔBarthel Index, discharged Barthel Index – Admission Barthel Index; >0 defined as improved and marked as 1; ≦0 defined as unimproved and marked as 0; ΔNIHSS, discharged NIHSS – Admission NIHSS; <0 defined as improved and marked as 1; ≧0 defined as unimproved and marked as 0. Improved of each EQ-5D scale (1, 0) represents patients got better or not after treatment.

In Table 2A, the degree of improvement before and after the post-acute care training is presented. From the paired sample *t*-test, it is evident that the admission and discharge Barthel Index values are significantly different (*P* = 0.039^*^) from each other (41.98 vs. 49.14). And the Index indicated an improvement in 71 patients from a total of 134 patients (52.99%).

**Table 2A:** Comparison of Barthel Index before and after admission.

	**Admission** **Barthel** **Index**	**Discharge Barthel** **Index**	**ΔBarthel** **Index**
Min.	0.00	0.00	−65.00
1st Qu.	20.00	30.00	−10.00
Median	37.50	50.00	5.00
Mean	41.98	49.14	7.16
3rd Qu.	55.00	65.00	25.00
Max.	100.00	100.00	100.00
T-test	*P* = 0.039^*^	Number of Unimproved (0)	63 (47.01%)
**N**	134	Number of Improved (1)	71 (52.99%)

ΔBarthel Index, Discharged Barthel Index – Admission Barthel Index; >0 defined as improved and marked as 1; ≦0 defined as unimproved and marked as 0;

* P ≤ 0.05.

In Table 2B, the paired sample *t*-test reveals that admission and discharge NIHSS values are significantly different (*P* = 0.037^*^) from each other (8.784 vs. 7.306). The NIHSS identified improvement in 68 patients from a total of 134 patients (50.75%).

**Table 2B:** Comparison of NIHSS before and after admission.

	**Admission NIHSS**	**Discharge NIHSS**	**ΔNIHSS**
Min.	0.000	0.000	−30.000
1st Qu.	4.000	3.000	−4.000
Median	8.000	7.000	−1.000
Mean	8.784	7.306	−1.478
3rd Qu.	12.000	11.000	1.000
Max.	30.000	21.000	12.000
T-test	*P* = 0.037^*^	Number of unimproved (0)	66 (49.25%)
N	134	Number of improved (1)	68 (50.75%)

ΔNIHSS = Discharged NIHSS – Admission NIHSS; <0 defined as improved and marked as 1; ≧0 defined as unimproved and marked as 0;

* P ≤ 0.05.

Table 3A shows the results of the independent samples *t*-test. It shows that DBP (92.370 vs. 86.957, *P* = 0.033), ipsilateral plaque index (3.158 vs. 2.295, *P* = 0.015), contralateral plaque index (3.888 vs. 2.535, *P* = 0.001), cholesterol (171.000 vs. 158.549, *P* = 0.045) and LDL (112.549 vs. 99.067, *P* = 0.027) are significant variables determining patient improvement in the Barthel Index. Similarly, contralateral CCA RI (0.781 vs. 0.756, *P* = 0.041), contralateral VA RI (0.828 vs. 0.770, *P* = 0.027) and contralateral plaque index (3.787 vs. 2.647, *P* = 0.006) are significant variables determining patient improvement in the NIHSS.

**Table 3A:** Important baseline variables for Barthel Index and NIHSS.

	**Barthel Index**			**NIHSS**		
	**Unimproved**	**Improved**	***P*** **value**	**Unimproved**	**Improved**	***P*** **value**
DBP	92.370	86.957	0.033^*^	91.769	87.529	0.071
Contra CCA RI	0.764	0.772	0.714	0.781	0.756	0.041^*^
Contra VA RI	0.808	0.787	0.242	0.828	0.770	0.027^*^
Ipsi Plaque Index	3.158	2.295	0.015^*^	3.030	2.470	0.082
Contra Plaque Index	3.888	2.535	0.001^**^	3.787	2.647	0.006^**^
Cholesterol	171.000	158.549	0.045^*^	166.348	163.823	0.367
LDL	112.549	99.067	0.027^*^	106.090	105.785	0.482

Independent Samples T-test. And mean of significant variables in each category (improved or not).

* P ≤ 0.05;

** P ≤ 0.01.

DBP, diastolic blood pressure; Ipsi, ipsilateral; Contra, contralateral; CCA, common carotid artery; VA, vertebral artery; RI, resistance index; LDL, low density lipoprotein.

Table 3B, illustrates the association between the baseline biochemistry/EQ-5D and the outcome assessment. The chi-square test of independent variables shows that mixed type hyperlipidemia (Yule's Q = 0.800, *P* = 0.014), self-care improvement (Yule's Q = 0.434, *P* = 0.026) and improvement of usual activities (Yule's Q = 0.513, *P* =0.032) are significant variables indicating improvement in the Barthel Index. Similarly, improvements in pain/discomfort (Yule's Q = 0.450, *P* = 0.026) and anxiety/depression (Yule's Q = 0.481, *P* = 0.006) are significant variables determining improvement in the NIHSS.

**Table 3B:** The association between baseline biochemistry/EQ-5D and outcome assessment.

	**Barthel Index**		**NIHSS**	
	***P*** **value**	**Yule's Q**	***P*** **value**	**Yule's Q**
Mixed type hyperlipidemia	0.014^*^	0.800	0.372	0.280
Self-care improved	0.026^*^	0.434	0.275	0.214
Usual activities improved	0.032^*^	0.513	0.166	0.331
Pain/discomfort improved	0.126	0.318	0.026^*^	0.450
Anxiety/depression improved	0.218	0.229	0.006^**^	0.481

Self-care improved is defined as 1. Usual Activities Improved is defined as 1. Pain/Discomfort Improved is defined as 1. Anxiety/Depression Improved is defined as 1. Chi-square Test of Independent. Yule's Q is a correlation coefficient, −1 ≦ Q ≦ 1.

* P ≤ 0.05;

** P ≤ 0.01.

Table 4 shows the result of univariate and multivariate logistic regression models of the Barthel Index. It shows that the probability of an improved Barthel Index for patients suffering from mixed-type hyperlipidemia was 8.855 times higher than for those who had no improvement in the Index (*P* = 0.041). Similarly, the probability of an improved Barthel Index for patients with self-care improvement was 2.492 times higher than for those who did not have an improved Barthel Index (*P* = 0.033). Likewise, the probability of an improved Barthel Index in patients with usual activities improvement was 3.054 times more than in those whose Barthel Index (*P* = 0.042) showed no improvement. In contrast, the probability of an improved Barthel Index in patients was 0.835 times less than in those who registered no improvement in the Barthel Index with every ipsilateral plaque index increasing (*P* = 0.025). The probability of an improved Barthel Index in patients was 0.819 times less than in those who showed no improvement in the Barthel Index for every contralateral plaque index increase (*P* = 0.005). In the multivariate stepwise logistic regression model, the probability of an improved Barthel Index for patients with usual activities improvement was 3.788 times more than those who had no improvement in the Barthel Index (*P* = 0.025), which indicates that patients with improved usual activities might score better on the Barthel Index. The probability of an improved Barthel Index in patients was 0.794 times less than in those who had no improvement of the Barthel Index for every contralateral plaque index increase (*P* = 0.002), which indicates that the higher the contralateral plaque index is, the less the patients improve. And the multivariate logistic regression model has 65% accuracy in prediction.

**Table 4:** Logistic regression model for Barthel Index assessment.

	**Univariate**		**Multivariate (stepwise)**	
	**OR**	***P*** **value**	**OR**	***P*** **value**
Mixed type hyperlipidemia	8.855	0.041^*^	7.842	0.062
Self-care improved	2.492	0.033^*^		
Usual activities improved	3.054	0.042^*^	3.788	0.025^*^
DBP	0.980	0.066		
Ipsi Plaque Index	0.835	0.025^*^		
Contra Plaque Index	0.819	0.005^**^	0.794	0.002^**^
Cholesterol	0.993	0.081		
LDL	0.991	0.044^*^	0.993	0.126

Self-care Improved is defined as 1. Usual Activities Improved is defined as 1.

* P ≤ 0.05;

** P ≤ 0.01.

DBP, diastolic blood pressure; Ipsi, ipsilateral; Contra, contralateral; LDL, low density lipoprotein.

Table 5 shows the results of the univariate and multivariate logistic regression models of NIHSS. The probability of improved NIHSS in patients with improved pain was 2.695 times more (*P* = 0.026), and in patients with improved anxiety was 2.752 times more (*P* = 0.010) than in those who showed no improvement in the NIHSS. In contrast, the probability of improved NIHSS for patients was 0.846 times less than for those who have no improvement of NIHSS for every contralateral plaque index increase (*P* = 0.015). In the multivariate stepwise logistic regression model, the probability of improved NIHSS for patients with anxiety improvement was 2.298 times more than for those who have no improvement of NIHSS (*P* = 0.045^*^), showing that patients with anxiety improvement might score better on the NIHSS. Similarly, the contralateral plaque index also showed statistically significant differences between improved and unimproved patients' NIHSS (*P* = 0.003). The multivariate logistic regression model has 65% accuracy in prediction.

**Table 5:** Logistic regression model for NIHSS assessment.

	**Univariate**		**Multivariate (stepwise)**	
	**OR**	***P*** **value**	**OR**	***P*** **value**
Pain improved	2.695	0.026^*^		
Anxiety improved	2.752	0.010^*^	2.298	0.045^*^
Contra CCA RI	0.023	0.085		
Contra VA RI	0.099	0.051	0.097	0.059
Contra Plaque Index	0.846	0.015^*^	0.870	0.003^*^

Pain improved defined as 1. Anxiety improved defined as 1.

* P ≤ 0.05.

Ipsi, ipsilateral; Contra, contralateral; CCA, common carotid artery; VA, vertebral artery; RI, resistance index.

**Figure 2:**
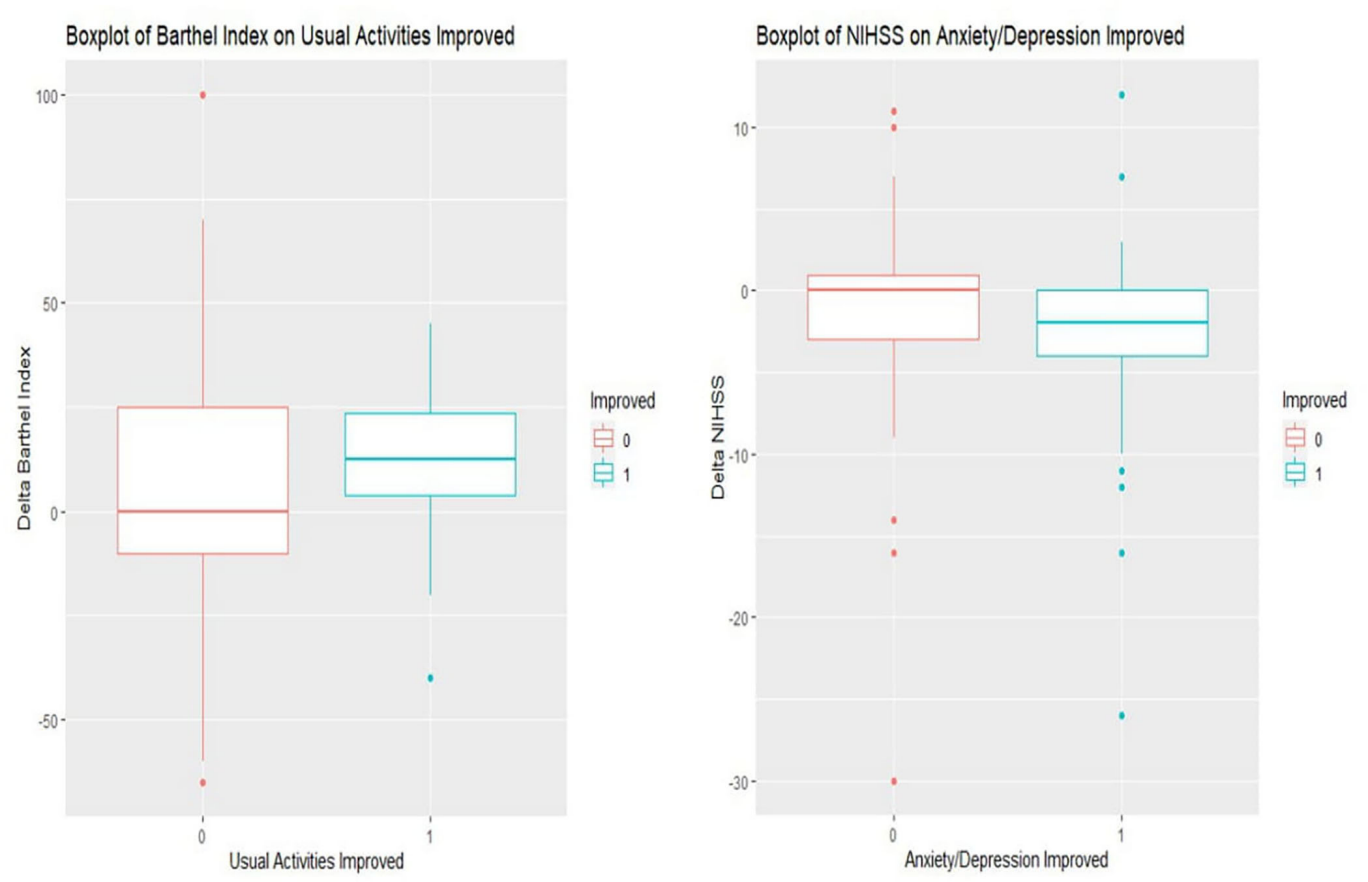
Boxplots of important domains of EQ-5D associated with NIHSS and Barthel Index scorings. In Barthel Index (left side of boxplot) (with usual activities improvement labeled as 1 and non-improvement as 0), it shows that patients with improved usual activities have a better Barthel Index than their counterparts after post-acute care rehabilitation (median of delta Barthel Index: 12.5 vs. 0.0, the more the better). In NIHSS (right side of boxplot) (with anxiety/depression improvement labeled as 1 while and non- improvement as 0), it reveals that patients with improved anxiety/depression show better NIHSS scorings than those without improvement (median of delta NIHSS: −2.0 vs. 0.0, the less the better). ΔBarthel Index = Discharge Barthel Index – Admission Barthel Index, >0 defined as improved and, ≦0 defined as unimproved; ΔNIHSS = Discharge NIHSS – Admission NIHSS, <0 defined as improved; ≧0 defined as unimproved.

Figure 2 displays the boxplot of outcome assessments on EQ-5Ds correlated with Barthel Index and NIHSS scorings. It showed that patients with improved usual activities had better Barthel Index scorings than those without improvement after post-acute care rehabilitation (median of delta Barthel Index: 12.5 vs. 0.0, where the higher the value, the better the assessment). The EQ-5D improvement also revealed that patients with improved anxiety/depression had better NIHSS scorings than those without improvement after rehabilitation (median of delta NIHSS: −2.0 vs. 0.0, where the lesser the value, the better the assessment).

**Figure 3:**
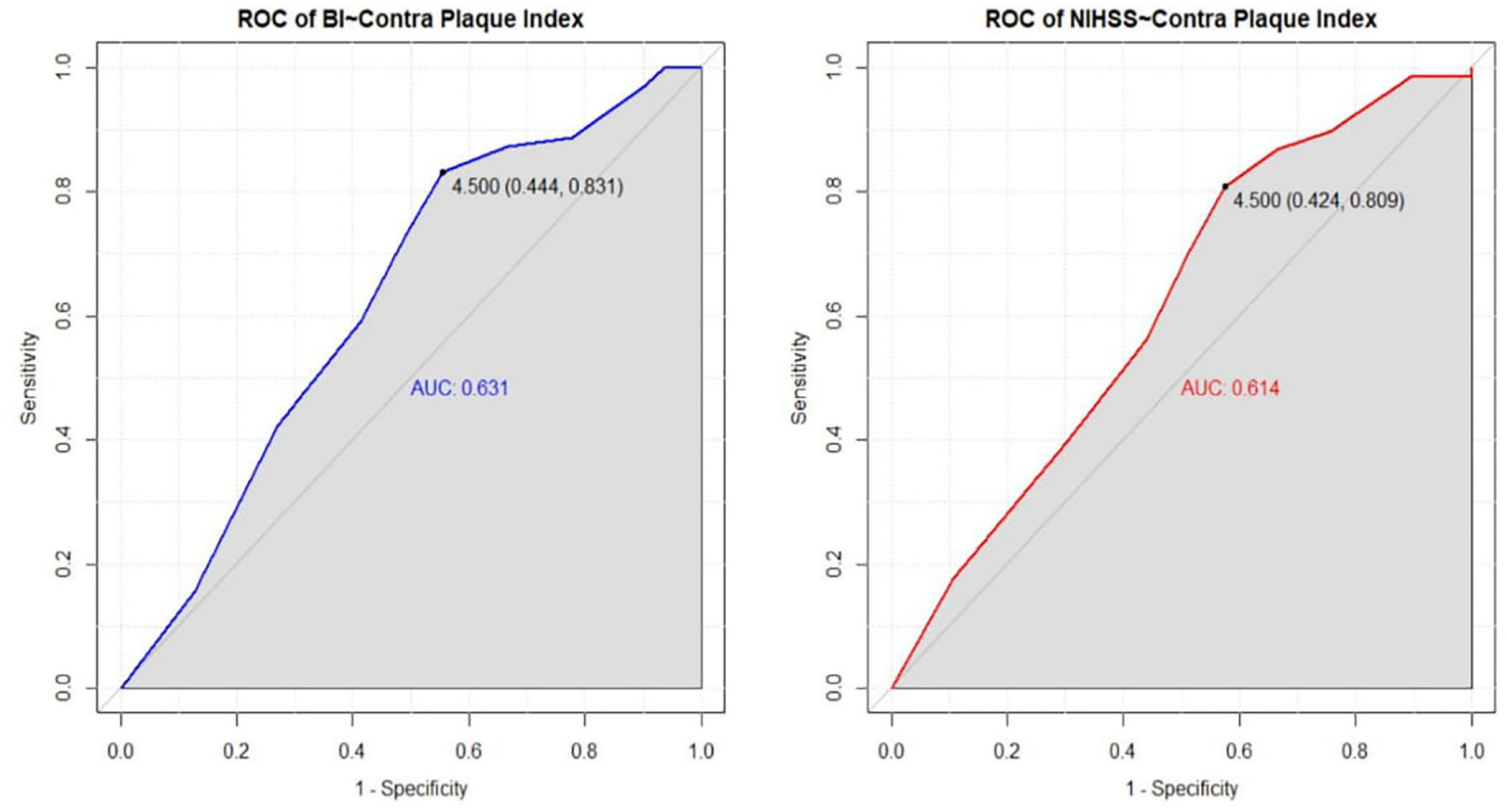
ROC curve of Contralateral Plaque Index from Barthel Index and NIHSS assessment. It demonstrated the result of the ROC curve of significant variable contralateral plaque index. In Barthel Index, the ROC curve finds the cut-off point of contralateral plaque index which is 4.5 with Youden's index (0.444, 0.831), and the area under the curve (AUC) indicating the overall accuracy of the degree of disability and the variables is 0.631. In NIHSS, the ROC curve finds the cut-off point of the contralateral plaque index which is 4.5 with Youden's index (0.424, 0.809), and the area under the curve (AUC) is 0.614. It reveals that the patient will not improve when the contralateral plaque index is over 4.5 in assessment outcomes, both Barthel Index and NIHSS.

Figure 3 demonstrates the result of the ROC curve of significant variable contralateral Plaque Index. In the Barthel Index, the ROC curve found the cut-off point of the contralateral plaque index which was 4.5 with Youden's index (0.444, 0.831), and the area under the curve (AUC) indicating that the overall accuracy of the degree of disability and the variables is 0.631. In NIHSS, the ROC curve found the cut-off point of the contralateral plaque index at 4.5 with Youden's index (0.424, 0.809), and the area under the curve (AUC) was 0.614. This reveals that patients will not improve when their contralateral plaque index is over 4.5 in assessment outcomes for both the Barthel Index and NIHSS.

## Discussion

With ischemic stroke being a major cause of death, much academic attention has been on the acute phase of management of ischemic stroke with atrial fibrillation. However, there has been less focus on the sub-acute and chronic phases of rehabilitative training programs that have been designed for the speedy recovery of the neurological and daily life activities of patients. Reviewing the current literature, it is possible to notice how the inclusion and exclusion criteria of aggressive intervention therapy namely, intravenous thrombolytic and/or intra-arterial (IA) thrombectomy treatments (3–5), and the cardiogenic origin induced index stroke episode have not been thoroughly investigated, as well as the long-term outcome for this type of stroke patients. For most patients with stroke and atrial fibrillation/flutter, doctors often prescribe new-generation anticoagulant medications for secondary stroke prevention. Clinicians also advise against intensive daily activities to prevent bumping the head accidentally or to be more alert to the presence of blood in the stools as a side effect of the anti-coagulant medication that causes blood thinning (26). These measures could discourage the patients from taking part eagerly in the rehabilitative process and recovery training. The PAC under this recovery scheme provides an intense training course, which usually lasts from one to two months and often plays a significant role in the transitional phase between a hospital stay and home stay (27). It also stresses the importance of the non-physical but equally important aspects of rehabilitation, through the EQ-5D, where the patient's emotional fluctuations, pain/discomfort, and more complex integrated daily life activity capacities are evaluated before being discharged. We believe this study is the first to analyze the outcomes of PAC among ischemic stroke patients with atrial fibrillation, and our findings could be significant for first-line clinicians focusing on the long-term recovery of functionalities in patients.

In the current study, the long-term mRS evaluation shows less substantial improvement compared to the NIHSS and Barthel Index (BI). This could be because the mRS is only grading the patient recovery functionality from 0 to 6, with lower coverage of other functional domains, while the NIHSS and BI are evaluating a larger number of variables and functions in both physical and non-physical domains. The other reason for this result might also be due to the follow-up period of the patient post-discharge being relatively short, whereas longer follow-ups might reflect more difference and perhaps the true functionality of recovery. However, the NIHSS in the current research shows nearly half of the enrolled cases demonstrated neurological defects recovery, especially limb weakness. To enhance muscle power recovery, more intense training is needed. The benefits of this program could appear as physical improvements on the NIHSS testing and may consequently also boost patients' confidence and motivation to continue the physical training processes.

The highlight of the current research is the inclusion of the EQ-5D evaluation as part of the outcome assessment process. In fact, other evaluating batteries have also been launched (such as MNA, FOIS, and IADL) but none of them show statistical significance except for the EQ-5D. It is Hanming Christian Hospital's protocol to use the above-mentioned batteries during the training process. It was found that anxiety/depression (30.99%) has a greater impact on the patient's long-term recovery. As this domain of the study is rather subjective, to be accurate and more objective during the evaluation process, the testing was carried out by case managers who could eventually compare their results to exclude any potential discrepancy. If there were any differences the attending physician was involved to assess and provide a final validation. The results from the testing revealed that carrying on with usual activities and self–care played an important role in the patient's recovery and correlated with the NIHSS and Barthel Index. EQ-5D was used to evaluate the patients and the findings suggest that the emotional stability of the individual and one's ability to complete a satisfactory recovery can be affected by mood swings. Low doses of antidepressant medications could reduce these instances and help those in need to continue the recovery process (28, 29). In addition, while NIHSS, BI, and mRS focus mostly on the left cerebral hemisphere, EQ-5D could reflect the partial functional recovery of the right cerebral side, therefore being complementary to previous outcome assessment tools. In the literature review (28–30), most studies had documented the anatomical distribution and difference, but few investigated emotional factors, which our research highlights.

This research reveals that plaque index (23) could potentially reflect the outcome and correlates well with the NIHSS and Barthel Index assessment batteries. It revealed that the higher its value, the worse the patient's outcome. It is our hospital protocol to calculate the plaque index of the carotid duplex examination testing from proximal and distal parts of the common carotid artery (CCA), carotid bulb, internal carotid artery (ICA) to external carotid artery (ECA) with the following grading: zero (non-plaque), 1 (less than one small plaque), 2 (one medium size or 2 more small plaques), 3 (one big plaque or multiple small plaques). Since these gradings might be somewhat subjective in the course of the evaluation, the hospital policy also adds intima-media thickness with >1 cm (either the right or left side), adding one point to the total summation to reflect the true genuine whole carotid systems situation and the quality of carotid extra cranial systems. Both NIHSS (AUC: 0.631) and BI (0.614) of the ROC and Youden shows the above cut-off point might hint that anything above 4.5 may lead to a worsening of long-term outcomes. However, none of the published literature mentioned this point, so this study is the first to discuss this finding. Moreover, we also noticed that most of the higher scored carotid systems had heterogeneous plaques in characteristics by B mode evaluation of carotid ultrasound testing. This type of plaque might lead to another stroke episode, which could potentially have a bleak outcome for the patient (31). It appears that in cases of a right hemisphere stroke if the contralateral side is functioning correctly the blood will flow to the symptomatic side. However, if the carotid system on the contralateral side is not working at optimum this could result in blood not flowing smoothly and normal blood flow is jeopardized. This was evidenced in this study as it was clear that patients with higher contralateral plaque index values demonstrated a poor neurological outcome, leading us to hypothesize that this may be due to damaged contralateral carotid flows not having the capability to either secure or salvage the symptomatic ipsilateral carotid system of the index stroke episode.

The underlying mixed-type hyperlipidemia in this research potentially played a significant role in correlating with the Barthel index long-term outcome assessment (Yule Q testing, with 0.8), as most of the included patients were elderly and were suffering from an underlying arrhythmia. If low-density lipoprotein (LDL) is elevated, the clinical outcome is even poorer, as compared to those without. This may be due to an increased cholesterol deposition on the already existing plaque formation making it more heterogeneous. Subsequently, it could cause the already formed plaque formation attached to the surface of the vessel wall to inflame further. This could then cause more debris in the bloodstream and the potential risk of bigger clots in either the extra-cranial or intracranial vasculature. In addition, this altered plaque could drift from the proximal to the distal part of the same blood vessel. This is especially true in the internal carotid artery and the subsequent intracranial middle cerebral artery. The plaque could then occult in the small arterioles in the distal branch arteries and induce a recurrent stroke emboli event. We also observed that a recurrent stroke not only occurs in the brain region but also in other human organs as well such as the heart or kidneys. It is therefore important to control the underlying cardiac rhythm and check the systemic lipid profile to prevent a secondary stroke and to favor better long-term recovery results.

The strength of this research can be found in different aspects: (1) the low degree of heterogeneity among the participants. Patients included in the study came from the surrounding local communities and shared the same ethnic group. Ischemic stroke patients with the same underlying atrial fibrillation/ flutter condition have not been thoroughly studied in the literature yet; (2) the technical consistency, as the same technician performed all the carotid duplex scans, together with the ability to compare each patient's functional scores one year after the treatment, which guaranteed an adequate time to also observe functional improvement; (3) patients were handled by the same facility and transferred to the affiliated hospital, with same management care and rehabilitation programs. The medical staff, nursing staff, and case manager traveled back and forth between the two hospitals to coordinate the needs of the patients and family members.

On the other hand, the major limitation of the current study is the relatively small sample size (*N* = 142) as well as the fact there was no placebo group for comparison. Besides, the homogeneity of the ethnic group can limit the general application of the findings. The follow-up of carotid duplex and trans-cranial color-coded duplex imaging (32) was also not documented to reflect the patients' cerebral blood flows condition, after being discharged. In addition, the long-term follow-up (up to 12 months or longer) of the NIHSS and BI has not been documented. The inter-evaluation battery tools comparison was not done to compare discrepancies. The right or left side of the cerebral lesions were not differentially documented, and the true genuine contribution of the stroke factor and subsequent intracranial flow condition could not be clearly depicted. The concomitant application of the drugs condition has not been recorded (antiplatelet or anticoagulant drugs or combined) (33, 34), as this might play a confounding factor that alters the long-term outcome of stroke patients.

## Conclusion

In the current study, nearly half of stroke patients showed favorable functional outcomes. The post-training outcome assessment correlates well with NIHSS and Barthel Index scores instead of mRS scoring. The importance of the patient's emotional stability in the post-stroke stage (anxiety /depression), along with the resumption of usual activities, which were discovered in the EQ-5D testing during the PAC program, are associated with and have a great impact on the patient receiving post-acute care rehabilitative strategy. Furthermore, the carotid duplex parameters obtained from the baseline testing upon admission to the neurological ward of Changhua Christian Hospital, including plaque index, might play a significant role in determining and differentiating the stroke patients' long-term functional outcome as well as aiding the clinical decision-making process.

## Data availability statement

The raw data supporting the conclusions of this article will be made available by the authors, without undue reservation.

## Ethics statement

The Institutional Review Board of Changhua Christian Hospital (CCH) approved this retrospective cohort study, which was performed in Changhua Christian Hospital and Hanming affiliated Christian Hospital. Written informed consent was waived due to decoding between original datasets and personal information, as well as approval from the IRB of CCH (#210713).

## Author contributions

Y-LH and S-YL: data collection, validation, manuscript writing, and bio-statistical analysis. Y-CT: bio-statistical analysis, manuscript writing, validation, and data proofing. S-CW, Y-WL, and Y-TS: data collection and validation. M-CC: manuscript preparation, bio-statistical analysis, data collection, and manuscript writing. C-HL: data validation. C-ML: study design, manuscript preparation, bio-statistical analysis, data collection, IRB preparation, and manuscript writing. All authors contributed to the article and approved the submitted version.

## Conflict of interest

The authors declare that the research was conducted in the absence of any commercial or financial relationships that could be construed as a potential conflict of interest.

## Publisher's note

All claims expressed in this article are solely those of the authors and do not necessarily represent those of their affiliated organizations, or those of the publisher, the editors and the reviewers. Any product that may be evaluated in this article, or claim that may be made by its manufacturer, is not guaranteed or endorsed by the publisher.
