# Supplementary material for: Functional outcome prediction of ischemic stroke patients with atrial fibrillation accepting post-acute care training
Source: Front Neurol. 2022 Sep 23;13:954212. doi: 10.3389/fneur.2022.954212 (PMC9539964; doi:10.3389/fneur.2022.954212)
Supplement: Supplementary file 1 [file Data_Sheet_1.docx]

**Appendix**

**Table A1. Comparison of EQ5D Mobility before and after admission**.

|  | Admission Mobility | Discharge Mobility | $\Delta$Mobility |
| --- | --- | --- | --- |
| Min. | 1.000 | 1.000 | -1.000 |
| 1st Qu. | 2.000 | 2.000 | 0.000 |
| Median | 2.000 | 2.000 | 0.000 |
| Mean | 2.035 | 1.858 | -0.177 |
| 3rd Qu. | 2.000 | 2.000 | 0.000 |
| Max. | 3.000 | 3.000 | 1.000 |
| T-test | p-value = 0.000* | Number of Unimproved (0) | 115 (81.56%) |
| N | 141 | Number of Improved (1) | 26 (18.44%) |

***Note*.** ∆Mobility = Discharged Mobility – Admission Mobility, <0 defined as improved and marked as 1, ≧0 defined as unimproved and marked as 0.

**Table A2. Comparison of EQ5D Self-care before and after admission.**

|  | Admission Self-care | Discharge Self-care | $\Delta$Self-care |
| --- | --- | --- | --- |
| Min. | 1.000 | 1.000 | -2.000 |
| 1st Qu. | 2.000 | 2.000 | -1.000 |
| Median | 2.000 | 2.000 | 0.000 |
| Mean | 2.291 | 2.028 | -0.262 |
| 3rd Qu. | 3.000 | 2.000 | 0.000 |
| Max. | 3.000 | 3.000 | 0.000 |
| T-test | p-value = 0.000* | Number of Unimproved (0) | 105 (74.47%) |
| N | 141 | Number of Improved (1) | 36 (26.53%) |

***Note*.** ∆Self-care = Discharged Self-care – Admission Self-care, < 0 defined as improved and marked as 1, ≧ 0 defined as unimproved and marked as 0.

**Table A3. Comparison of EQ5D Usual Activities before and after admission.**

|  | Admission Usual Activities | Discharge Usual Activities | $\Delta$Usual Activities |
| --- | --- | --- | --- |
| Min. | 1.000 | 1.000 | -2.000 |
| 1st Qu. | 2.000 | 2.000 | 0.000 |
| Median | 2.000 | 2.000 | 0.000 |
| Mean | 2.234 | 2.071 | -0.163 |
| 3rd Qu. | 3.000 | 2.000 | 0.000 |
| Max. | 3.000 | 3.000 | 0.000 |
| T-test | p-value = 0.006* | Number of Unimproved (0) | 121 (85.82%) |
| N | 141 | Number of Improved (1) | 21 (14.18%) |

***Note*.** ∆ Usual Activities = Discharged Usual Activities - Admission Usual Activities, <0 defined as improved and marked as 1, ≧0 defined as unimproved and marked as 0.

**Table A4. Comparison of EQ5D Pain/Discomfort before and after admission.**

|  | Admission Pain/Discomfort | Discharge Pain/Discomfort | $\Delta$Pain/Discomfort |
| --- | --- | --- | --- |
| Min. | 1.000 | 1.000 | -1.000 |
| 1st Qu. | 2.000 | 2.000 | 0.000 |
| Median | 2.000 | 2.000 | 0.000 |
| Mean | 2.035 | 1.858 | -0.177 |
| 3rd Qu. | 2.000 | 2.000 | 0.000 |
| Max. | 3.000 | 3.000 | 1.000 |
| T-test | p-value = 0.000* | Number of Unimproved (0) | 112 (79.43%) |
| N | 141 | Number of Improved (1) | 29 (20.57%) |

***Note*.** ∆Pain/Discomfort = Discharged Pain/Discomfort – Admission Pain/Discomfort, < 0 defined as improved and marked as 1, ≧ 0 defined as unimproved and marked as 0.

**Table A5. Comparison of EQ5D Anxiety/Depression before and after admission.**

|  | Admission Anxiety/Depression | Discharge Anxiety/Depression | $\Delta$Anxiety/Depression |
| --- | --- | --- | --- |
| Min. | 1.000 | 1.000 | -2.000 |
| 1st Qu. | 2.000 | 1.000 | -1.000 |
| Median | 2.000 | 2.000 | 0.000 |
| Mean | 2.078 | 1.738 | -0.219 |
| 3rd Qu. | 2.000 | 2.000 | 0.000 |
| Max. | 3.000 | 3.000 | 1.000 |
| T-test | p-value = 0.000* | Number of Unimproved (0) | 97 (68.79%) |
| N | 141 | Number of Improved (1) | 44 (31.21%) |

***Note.*** ∆Anxiety/Depression = Discharged Anxiety/Depression – Admission Anxiety/Depression, < 0 defined as improved and marked as 1, ≧ 0 defined as unimproved and marked as 0.

**IRB certificate**
